# Anticipating impact of implementing PCV20 or PCV21 vaccines for older adults in the immunisation programme for invasive pneumococcal disease, using nationwide surveillance data, Israel, 2009 to 2024

**DOI:** 10.2807/1560-7917.ES.2026.31.15.2500589

**Published:** 2026-04-16

**Authors:** Romy Rieger, Dafna Yahav, Ili Margalit, Anat Wieder-Finesod, Hovav Azulay, Nesrin Ghanem-Zoubi, Alaa Atamna, Yehudit Schindler, Hiba Abu Zayyad, Ron Dagan, Gili Regev-Yochay, on behalf of the Israeli Adult Pneumococcal Invasive Disease (IAIPD) group

**Affiliations:** 1 Infection control and prevention unit, Sheba Medical Center, Ramat Gan, Israel; 2 Faculty of medical & health sciences, Tel Aviv University, Ramat-Aviv, Tel Aviv, Israel; 3 Infectious Diseases Unit, Sheba Medical Center Ramat Gan, Israel; 4 Infectious Disease Institute, Soroka University Medical Center, Beer Sheba, Israel; 5 The Faculty of Health Sciences, Ben-Gurion University of the Negev, Beer Sheba, Israel; 6 Infectious Diseases Institute, Rambam Health Care Campus, Haifa, Israel; 7 The Ruth and Bruce Rappaport Faculty of Medicine, Technion, Israel Institute of Technology, Haifa, Israel; 8 Infectious Disease Unit, Rabin Medical Center, Beilinson Hospital, Petah-Tikva, Israel; 9 Microbiology Laboratory, Mayanei Hayeshua Medical Center, Bnei Brak, Israel; 10 Infectious Diseases Unit, The Baruch Padeh Medical Center Poriya, Tiberias, Israel; 11 The Shraga Segal Dept. of Microbiology, Immunology and Genetics, Faculty of Health Sciences of the Ben-Gurion University of the Negev, Beer-Sheva, Israel; 12 The members of the Israeli Adult Pneumococcal Invasive Disease (IAIPD) group and the Microbiology Group are acknowledged at the end of the article

**Keywords:** Pneumococcal conjugate vaccine, Invasive pneumococcal disease, PCV20, PCV21, Vaccine impact

## Abstract

**BACKGROUND:**

Pneumococcal conjugate vaccines (PCVs) have reduced vaccine-type (VT) invasive pneumococcal disease (IPD) in older adults through direct and indirect effects. However, non-vaccine-type serotypes have emerged. Since the recently licensed PCV20 and PCV21 vaccines differ in serotype composition, epidemiological data are essential to guide adult vaccination policy.

**AIM:**

We aimed to assess serotype-specific IPD dynamics in older adults in Israel and evaluate the potential impact of implementing either the PCV20 or PCV21 vaccine in the adult National Immunisation Programme (NIP).

**METHODS:**

In a national active IPD surveillance study in Israel, 2009–2024, on adults aged ≥ 65 years, IPD incidence per 100,000 population was assessed by age group, individual serotype and VT group.

**RESULTS:**

We recorded 3,553 IPD episodes. All-IPD incidence was relatively stable between 2014/15 and 2023/24, excluding the COVID-19 period. In the late PCV13 period (2014–2019, 1,210 IPD episodes) and late/post COVID-19 pandemic (2021-2024, 608 episodes) periods, incidence of serotypes unique to PCV21 (VT21-only) was consistently higher than that of those unique to PCV20 (VT20-only). During 2022–2024, VT21-only IPD constituted 25.5% of all-IPD cases compared with 10.9% for VT20-only. Of the VT20-only serotypes, serotype 14 showed the highest late/post-pandemic incidence (1.76 per 100,000).

**CONCLUSIONS:**

The PCV21 vaccine currently demonstrates broader serotype coverage than PCV20 among older adults in Israel. However, the spectrum of serotypes only partially overlap. Potential further serotype-specific dynamics following PCV20 implementation in the paediatric NIP and unknown actual effectiveness of newly introduced vaccine serotypes make it difficult to accurately predict impact following implementation.

**Key public health message**

**What did you want to address in this study and why?**

Invasive pneumococcal disease (IPD) persists in older adults despite long-standing vaccination programmes. The recent licensure of two expanded-spectrum pneumococcal conjugate vaccines, PCV20 and PCV21, raises a dilemma regarding the optimal vaccine for adults. While PCV21 appears to potentially have a broader serotype coverage, real-world epidemiological evidence is essential to determine the clinical and public health impact.

**What have we learnt from this study?**

Using 15 years of national surveillance data on IPD in adults aged ≥ 65 years from Israel, we found that the PCV21 vaccine currently provides broader potential coverage. However, certain serotypes unique to the PCV20 vaccine remain notable contributors to disease burden and have even recently increased.

**What are the implications of your findings for public health?**

Our comprehensive analysis provides timely insights into the circulating serotypes that caused IPD in the late/post COVID-19 pandemic period (2021/22–2023/24). This information can be used to help facilitate decision-making at the public health level by anticipating the potential impact of implementing either the PCV20 or PCV21 vaccine in the adult National Immunisation Programme.

## Introduction

The implementation of pneumococcal conjugate vaccines (PCVs) in paediatric immunisation programmes resulted in a substantial decline in invasive pneumococcal disease (IPD) caused by pneumococcal conjugate vaccine-type (VT) serotypes worldwide [1-3]. However, this decline has been partly offset by the increase or emergence of non-vaccine-type serotypes (NVT), also termed serotype replacement [1]. While the overall benefits of implementing PCVs have been most evident in children, they have also been extended to adults through indirect (herd) protection [4,5]. However, in the pre-vaccine era, VT serotypes were found to account for only 40–50% of all IPD episodes in adults, particularly in immunocompromised individuals and older adults [5,6]. Therefore, a 70% decline in VT-IPD caused by indirect protection resulted in only a modest overall reduction in IPD [5,6].

To attempt to improve this modest reduction in IPD, many countries, including Israel, have adopted direct immunisation policies for at-risk adults, defined as older adults (≥ 65 years) and younger immunocompromised adults, prioritising vaccines with broader serotype coverage. Until recently, Israel's strategy consisted of administrating either the 23-valent polysaccharide pneumococcal vaccine (PPV23), the PCV vaccine (PCV7 was introduced in 2009 and replaced by PCV13 in 2010), or both. However, despite the success of PPV23 implementation among older adults (vaccine uptake of > 70%) and relatively wide use of the combined PCV/PPV23 vaccine among immunocompromised individuals, replacement with NVT-IPD among these populations has been considerable, possibly due to the relatively narrow serotype spectrum of the PCVs and the inherent limitation of the pure polysaccharide vaccine [5].

Recently, two expanded-spectrum PCVs have been licensed for adults: PCV20 and PCV21 [7,8]. The United States (US) Advisory Committee on Immunization Practices (ACIP) recommends either the PCV20 or PCV21 vaccine or the combination of the PCV15 and PPSV23 vaccines in 2024, without prioritising any of the options [9].

In Israel, The PCV7 and PCV13 vaccines were introduced into the paediatric National Immunisation Programme (NIP) in July 2009 and November 2010, respectively, administered at 2, 4 and 12 months. By 2014, the third PCV13 dose uptake reached ca 90%. The PPV23 vaccine uptake among adults aged 65 years and older has been 76–79% in Israel since 2012 (data provided by the Israel MOH). In July 2023, the PCV20 vaccine was introduced for adults in Israel, replacing PPV23 due to its large overlap with serotypes covered by the PPV23 vaccine. The PCV20 vaccine covers 20 serotypes including all 13 serotypes covered by the PCV13 vaccine (VT13) plus seven additional serotypes (VT20–13), widely overlapping with the serotypes covered by the PPV23 vaccine and thus eliminating the need for PPV23. The PCV21 vaccine for use in adults covers 21 serotypes including four serotypes covered by the PCV13 vaccine, all PCV20–13 VT serotypes and 10 additional serotypes (VT21-only) not covered by the PCV20 vaccine. While the real-world impact of the PCV20 and PCV21 vaccines has yet to be established, the subsequent licensure of PCV21 raises a dilemma regarding the optimal choice of PCV in adult vaccination programmes. In contrast to PCV20, adopting PCV21, which lacks nine of the serotypes covered by PCV13, would largely rely on indirect protection against these serotypes from immunised children.

We aimed to assess the potential impact on IPD among adults aged 65 years or older from implementing either the PCV20 or PCV21 vaccine in the Israeli adult NIP. For this purpose, we used data collected as part of the ongoing Israel Adult Invasive Pneumococcal Disease (IAIPD) project, a nested project within the Israel National Immunisation Programme (IsraNIP) active, nationwide surveillance [6,10].

## Methods

### Setting, study population and study period

The IAIPD project is an ongoing nationwide active, population-based surveillance of IPD in adults (≥ 18 years) in Israel. It is a nested project within the surveillance on IPD covering all ages (IsraNIP), initiated in July 2009.

The current analysis covers a 15-year period (July 2009 to June 2024).

The study population was defined as adults aged 65 years and older, referred to as older adults. During the study period, this population grew from 0.7 million (2009) to 1.3 million (2023) as determined by the Israeli Central Bureau of Statistics (CBS) [11].

#### Surveillance system

Detailed methodologies for the IAIPD and IsraNIP surveillance programmes have been published previously [6,12]. Briefly, several isolate collecting methods were carried out; all invasive *Streptococcus pneumoniae* isolates are required to be reported and sent to the Ministry of Health (MOH) reference laboratory by law. In addition to this passive surveillance, an active surveillance using a capture-recapture method, where the IAIPD representative in each of the 27 laboratories that perform blood and CSF cultures is contacted on a weekly basis by the study headquarters (at the Soroka University Medical Center) as described previously [12]. Thus, we were able to minimise the risk of missing IPD cases.

Local investigators also reported the specific IPD clinical syndrome for each case, including meningitis, bacteraemic pneumonia, other IPD sources (bacteraemia with mastoiditis, etc.) and bacteraemia only (without apparent focus).

#### Case definition

Invasive pneumococcal disease episodes were defined by the isolation of *S. pneumoniae* from blood or cerebrospinal fluid (CSF). Since policies regarding aspirations from other sterile sites vary largely between medical centres, positive cultures from other sterile sites (i.e. joint, pleural fluid, peritoneal fluid) were excluded [12]. Diagnoses based solely on non-culture methods (PCR, antigen testing, gram stain or clinical diagnosis only) were also excluded.

### Laboratory testing

Serotyping was performed by the laboratory at the surveillance study headquarters using Quellung reaction (Staten Serum Institute, Copenhagen, Denmark).

### Data analysis

Pneumococcal serotypes were grouped according to vaccine coverage as follows: (i) the nine serotypes included in PCV20 but not in PCV21 (1, 4, 5, 6B, 9V, 14, 18C, 19F, 23F (VT20-only)), (ii) the 10 serotypes included in PCV21 but not in PCV20 (9N, 15A, 16F, 17F, 20, 23A, 23B, 24F, 31, 35B (VT21-only)), (iii) the 11 serotypes covered by both vaccines (3, 6A 7F, 8, 10A, 11A, 12F, 15B/C 19A, 22F, 33F (VT20 and 21)), and (iv) serotypes not covered by either PCV20 or PCV21 (NVT) [5,7] (serotype coverage comparison of PCV13, PCV20 and PCV21 vaccines are depicted in Supplementary Figure S1). Since serotypes 15B and 15C are cross-reactive and currently difficult to differentiate, we defined them as a single common serotype covered by both PCV20 and PCV21 vaccines, and for the purpose of this analysis we assumed that both vaccines have similar effectiveness against each of these two serotypes. We also assumed that although both PCVs contain serotype 3, their effectiveness against this serotype may be limited [5,13]. Accordingly, we also assessed serotype 3 separately, not grouped among the common vaccine serotypes. Although serotype 6C is affected by the immune response to serotype 6A and thus could be considered as a vaccine-related stereotype, we opted to not include it as a vaccine serotype as we did not expand our discussion to the potential impact on vaccine-related serotypes. Furthermore, serotype 6A is included in all discussed vaccines, thus no difference between them is expected with regard to potential impact on serotype 6C.

The study period was divided into the following timeframes (with each study year spanning from July to June): (i) early PCV7/13 (2009/10–2013/14), (ii) late PCV13 (2014/15–2018/19), (iii) COVID-19 pandemic (2019/20–2020/21), and (iv) late/post COVID-19 pandemic (2021/22–2023/24).

Since IPD rates during the late/post COVID-19 pandemic period returned to the pre COVID-19 pandemic rates observed during the late PCV13 period, we considered these two periods collectively as the 'stable period'. For some of the analyses we examined the most recent 2 years of the study (2022/23–2023/24) separately, acknowledging that despite their apparent similarity to the earlier stable period, they may reflect ongoing post-pandemic emerging trends that have yet to be fully captured.

Annual incidence rates (IRs) per 100,000 population were calculated for both serotype groups related to each vaccine and individual serotypes by dividing the number of IPD cases by the total age group population for each study year (age groups used: 65–74 years, 75–84 years and ≥ 85 years). Population denominators were derived from the Israeli CBS national population registry [11], based on the population size for the previous study year. Descriptive figures were drawn to illustrate the temporal dynamics of IPD caused by different serotype groups or single relevant serotypes throughout the 15-year study period. Incidence rate ratios (IRR) and Poisson 95% confidence intervals (CI) were calculated to compare the IRs of VT21-only IPD to those of VT20-only IPD, and to compare the overall potential coverage of the PCV21 vs PCV20 vaccines (i.e. VT21 vs VT20) during the stable period. In addition, IRRs were calculated to assess temporal changes in IPD incidence, both for all-IPD and by specific serotype groupings.

## Results

Between July 2009 and June 2024, a total of 3,553 IPD episodes were recorded among older adults in Israel, of which 608 occurred in the late/post COVID-19 pandemic years. Serotype was available for 3,407 (95.9%) of the isolates.

### Serotype dynamics over the entire surveillance period

**Figure 1:**
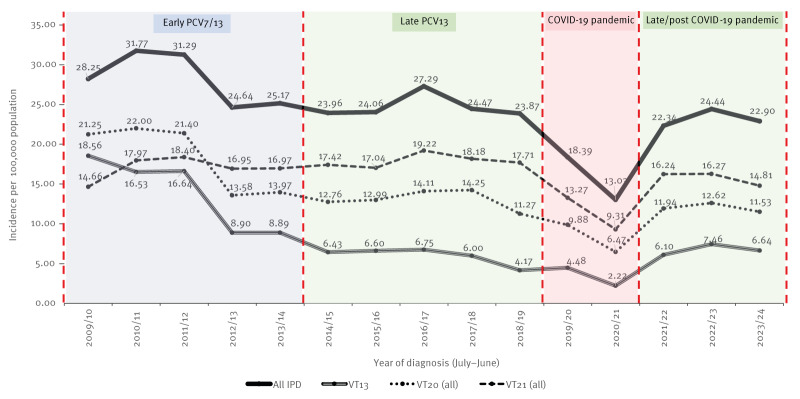
Annual incidence (per 100,000 population) of all invasive pneumococcal disease episodes and invasive pneumococcal disease episodes caused by serotypes covered by PCV13, PCV20 and PCV21 vaccines in adults 65 years and older, Israel, 2009–2024 (n = 3,553 invasive pneumococcal disease episodes)

IPD: invasive pneumococcal disease; PCV: pneumococcal conjugate vaccine; VT: pneumococcal conjugate vaccine serotypes.

Following the introduction of the PCV7 and subsequently PCV13 vaccines in the paediatric NIP, a decline of 15% (IRR: 0.85; 95% CI: 0.70–1.03) in all-IPD incidence among older adults was observed, from an IR (per 100,000) of 28.3 in 2009/10 to 24.0 in 2014/15, reaching a temporary plateau (stable period) (Figure 1). The decrease in all-IPD episodes was mostly attributable to a 65% decline (IRR: 0.35; 95% CI: 0.25–0.47) in overall VT13 serotypes, but was partly offset by the increase in NVT13 serotype episodes (serotype replacement) (Figure 1). During the late PCV13 period, PCV13 VT serotypes (VT13) accounted for 24.0% of all IPD episodes (IR: 5.95/100,000), while PCV20 VT serotypes (VT20) accounted for 52.8% (IR: 13.06/100,000) and PCV21 VT serotypes (VT21) accounted for 72.5% (IR: 17.93/100,000) (Table). During this period, VT20-only and VT21-only accounted for 8.1% and 27.8%, respectively (Table).

**Table:** Characteristics of all invasive pneumococcal disease episodes and invasive pneumococcal disease episodes caused by serotypes covered by the PCV13, PCV20 and PCV21 vaccines, as well as those unique to the PCV20 and PCV21 vaccines, (VT20-only and VT21-only), among adults aged 65 years or older, Israel, 2009–2024

Characteristics	Study period (year of diagnosis)				
	Early PCV7/13 (Jul 2009–Jun 2014)	Late PCV13 (Jul 2014–Jun 2019)	COVID-19 pandemic (Jul 2019–Jun 2021)	Last two years post COVID-19 pandemic (Jul 2022–Jun 2024)	Stable period^c^
All-IPD IR per 100,000 (n cases)	28.11 (n = 1,127)	24.73 (n = 1,210)	15.67 (n = 348)	23.65 (n = 608)	24.09 (n = 2,078)
VT13 IPD IR per 100,000 (n cases)	13.70 (n = 549)	5.95 (n = 291)	3.33 (n = 74)	7.04 (n = 181)	6.29 (n = 543)
VT13 IPD Proportion of all IPD (%)	48.71	24.05	21.26	29.77	26.13
VT20 IPD IR per 100,000 (n cases)	18.26 (n = 732)	13.06 (n = 639)	8.15 (n = 181)	12.06 (n = 310)	12.61 (n = 1,088)
VT20 IPD Proportion of all IPD (%)	64.95	52.81	52.01	50.99	52.36
VT21 IPD IR per 100,000 (n cases)	17.01 (n = 682)	17.93 (n = 877)	11.26 (n = 250)	15.52 (n = 399)	16.98 (n = 1,465)
VT21 IPD Proportion of all IPD (%)	60.51	72.48	71.84	65.62	70.50
IRR VT21/VT20 (95% CI)	0.93 (0.84–1.03)	1.37 (1.24–1.52)	1.38 (1.14–1.67)	1.29 (1.11–1.49)	1.35 (1.24–1.46)
VT20-only^a^ IPD IR (n cases)	6.94 (n = 278)	2.00 (n = 98)	1.35 (n = 30)	2.57 (n = 66)	2.11 (n = 182)
VT20-only^a^ IPD Proportion of all IPD (%)	24.67	8.10	8.62	10.86	8.76
VT21-only^b^ IPD IR (n cases)	5.69 (n = 228)	6.87 (n = 336)	4.46 (n = 99)	6.03 (n = 155)	6.48 (n = 559)
VT21-only^b^ IPD Proportion of all IPD (%)	20.23	27.77	28.45	25.49	26.90
IRR VT21-only / VT20-only (95% CI)	0.82 (0.69–0.98)	3.43 (2.74–4.30)	3.30 (2.20–4.97)	2.35 (1.76–3.13)	3.07 (2.60–3.63)

CI: confidence interval; IPD: invasive pneumococcal disease; IR: incidence rate; IRR: incidence rate ratio; PCV: pneumococcal conjugate vaccine; VT: pneumococcal conjugate vaccine serotype.

^a^ VT20-only includes the serotypes included in the PCV20 but not in the PCV21 vaccine.

^b^ VT21-only includes the serotypes included in the PCV21 but not in the PCV20 vaccine.

^c^ Stable period: late PCV13 (2014/15–2018/19) and late/post COVID-19 pandemic (2021/22–2023/24).

During the COVID-19 pandemic, a significant decline in all-IPD incidence was observed, from 23.9 per 100,000 in the year preceding the pandemic (2018/19) to 13.0 in the COVID-19 peak year (2020/21) (IRR: 0.55; 95% CI: 0.45–0.67) (Figure 1). However, the proportions of serotypes covered by VT13, VT20 and VT21 did not differ significantly when comparing the COVID-19 peak year to all other stable period years (Figure 1). Incidence rates, proportions and corresponding IRRs by vaccine serotype groups are shown in Supplementary Table S1. Following the pandemic, IPD incidence returned to pre-pandemic rates by 2022, reaching 22.9 cases per 100,000 population in 2023/24 (Figure 1). The overall potential coverage of the PCV21 vaccine compared with that of the PCV20 vaccine was 1.37 (95% CI: 1.24–1.52) and 1.29 (95% CI: 1.11–1.49) times higher than that of the PCV20 vaccine during the late PCV13 and late/post COVID-19 pandemic periods, respectively (Table).

### Invasive pneumococcal disease incidence caused by PCV20 and PCV21 vaccine-type serotypes during the stable period

**Figure 2:**
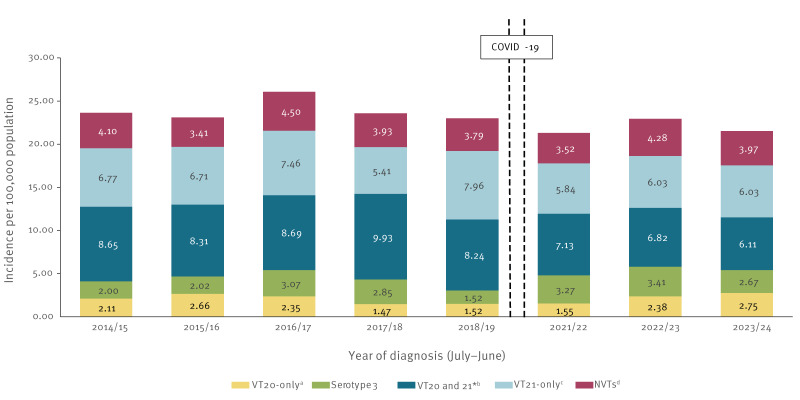
Annual invasive pneumococcal disease incidence per 100,000 population caused by specific vaccine- and non-vaccine-type serotypes in adults 65 years and older during the ‘stable period’ (late PCV13 period 2014/15–2018/19 and late/post COVID-19 pandemic periods 2021/22–2023/24), Israel (n = 2,078 invasive pneumococcal disease episodes)

IPD: invasive pneumococcal disease; NVT: non-pneumococcal conjugate vaccine serotypes; PCV: pneumococcal conjugate vaccine; VT: pneumococcal conjugate vaccine serotypes.

^a^ VT20-only includes the serotypes included in the PCV20 but not in the PCV21 vaccine.

^b^ VT20 and 21 include the serotypes covered by both the PCV20 and PCV21 vaccines, excluding serotype 3.

^c^ VT21-only includes the serotypes included in the PCV21 but not in the PCV20 vaccine.

^d^ NVT includes all serotypes not included in either the PCV20 or PCV21 vaccine.

All-IPD incidence was relatively stable from 2014/15 to 2023/24, excluding the COVID-19 pandemic period, referred to as the stable period (Figure 2). Yet, during the late PCV13 period, VT20-only and serotype 3 decreased somewhat (from 2.11 and 2.0/100,000 in 2014/15 to 1.52 and 1.52/100,000, in 2018/19, respectively) (Figure 2). However, in the last study year (2023/24), they increased to 2.75 (VT20-only) and 2.67 per 100,000 (serotype 3) (Figure 2).

The IRR of the mean annual incidence of VT21-only compared with that of the VT20-only during the late PCV13 period was 3.43 (95% CI: 2.74–4.30), while in the last 2 years post COVID-19 pandemic (2022–2024), it decreased to 2.35 (95% CI: 1.76–3.13). Thus, during the 2 most recent years of the study (2022–2024), VT21-only covered 25.5% of all-IPD cases vs 10.9% covered by VT20-only (Table).

### Leading serotypes in the late/post COVID-19 pandemic period

**Figure 3:**
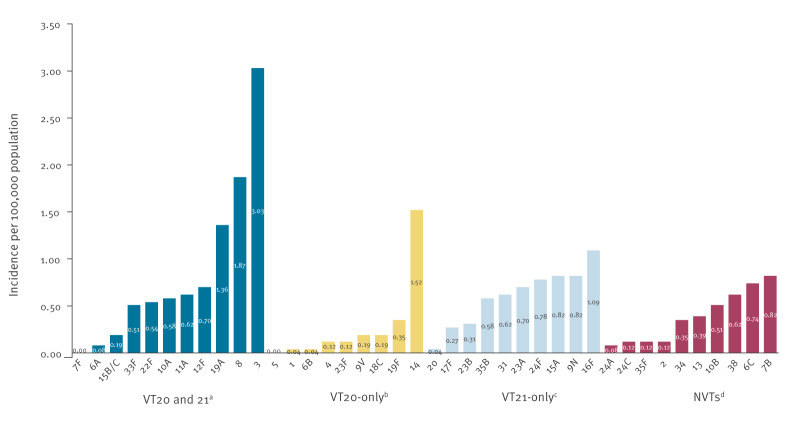
Incidence of invasive pneumococcal disease per 100,000 population caused by specific vaccine- and non-vaccine-type serotypes in adults 65 years or older during the late/post COVID-19 pandemic period, Israel, July 2022–June 2024

IPD: invasive pneumococcal disease; NVT: non-pneumococcal conjugate vaccine serotypes; VT: pneumococcal conjugate vaccine serotypes.

^a^ VT20 and 21 include the serotypes covered by both the PCV20 and PCV21 vaccine.

^b^ VT20-only includes the serotypes included in the PCV20 but not in the PCV21 vaccine.

^c^ VT21-only includes the serotypes included in the PCV21 but not in the PCV20 vaccine.

^d^ NVT includes all serotypes not included in either the PCV20 or PCV21 vaccine.

In the most recent 2 years of the study (2022–2024), the five most common serotypes were: 3 (IR: 3.03/100,000; proportion of all IPD: 12.8%), 8 (IR: 1.87/100,000; proportion of all IPD: 7.9%), 14 (IR: 1.52/100,000; proportion of all IPD: 6.4%), 19A (IR: 1.36/100,000; proportion of all IPD: 5.8%) and 16F (IR: 1.09/100,000; proportion of all IPD: 4.6%) (Figure 3, Supplementary Table S2 for IR and proportions of the most common individual serotypes, 2022–2024). The dynamics of these five serotypes during the years studied are depicted in Supplementary Figures S2–S6.

The most common VT20-only serotype in the late/post COVID-19 pandemic period was 14, which was also the only emerging serotype in this group (Figure 3, Supplementary Figure S4 shows the annual incidence dynamics of serotype 14 throughout the study period), followed by serotype 19F (IR: 0.35/100,000) (Figure 3, Supplementary Figure S7 shows the annual incidence dynamics of serotype 19F throughout the study period). Other serotypes in this group, including 18C, 9V and 23F, continued to circulate in the late/post COVID-19 pandemic period but to a lower degree (Figure 3). Serotype 4 maintained a low and stable incidence in our population (Supplementary Figure S8 shows the annual incidence dynamics of serotype 4 throughout the study period).

**Figure 4:**
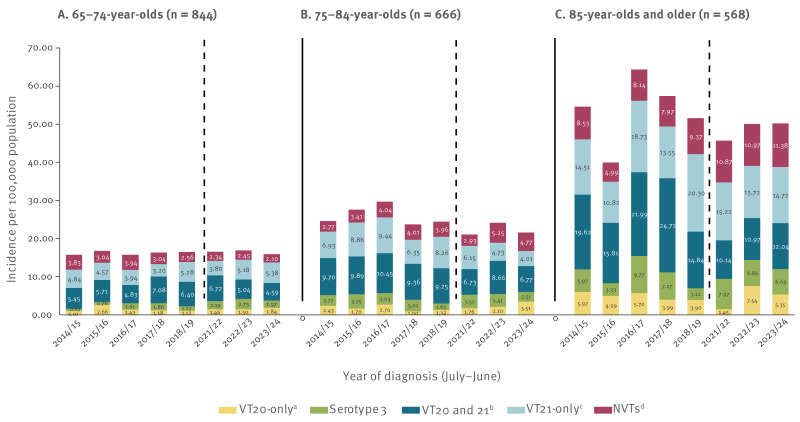
Annual invasive pneumococcal disease incidence per 100,000 population caused by specific vaccine- and non-vaccine-type serotypes in adults 65 years and older during the ‘stable period’ (late PCV13 period 2014/15–2018/19 and late/post COVID-19 pandemic periods 2021/22–2023/24), Israel (n = 2,078 invasive pneumococcal disease episodes)

IPD: invasive pneumococcal disease; NVT: non-pneumococcal conjugate vaccine serotypes; PCV: pneumococcal conjugate vaccine; VT: pneumococcal conjugate vaccine serotypes.

^a^ VT20-only includes the serotypes included in the PCV20 but not in the PCV21 vaccine.

^b^ VT20 and 21 include the serotypes covered by both PCV20 and PCV21 vaccines, excluding serotype 3.

^c^ VT21-only includes the serotypes included in the PCV21 but not in the PCV20 vaccine.

^d^ NVT includes all serotypes not included in either PCV20 or PCV21 vaccine.

The IR of serotype 3 did not change significantly over the full study period, remaining at ca 3.0 per 100,000 population throughout (Figure 2, Supplementary Figure S2 shows the annual incidence dynamics of serotype 3 throughout the study period), accounting for 12.8% of all serotypes in these years (Supplementary Table S2). The temporal pattern of serotype 3 persistence was demonstrated across all age groups (65–74, 75–84 and ≥ 85 years), with the 85 years and older group having the highest IRs and larger fluctuations over time, reaching up to 6.86 per 100,000 in recent years (Figure 4).

Dashed lines indicate the COVID-19 pandemic period 2019─2021.

## Discussion

In this study we have shown that both the PCV20 and PCV21 vaccines are expected to expand the range of preventable IPD episodes due to their coverage of additional serotypes beyond those in PCV13 vaccine. The two vaccines are not fully interchangeable due to only a partial overlap in their serotype content. Determining which vaccine could provide the best protection for the adult population aged 65 years and older should be based on epidemiological data and programmatic decisions, but there is still not enough information on several factors that may play an important role in the selection process. These include, among other potential factors, the lack of effectiveness data for all newly included serotypes, limited understanding of the immunologic response to some serotypes such as serotype 3 or serotype 15B/C, the potential loss of protection against certain serotypes due to the discontinuation of the PPV23 vaccination programme and the possible emergence of selective pressure on specific serotypes in the community following the introduction of extended-serotype PCVs to the paediatric NIP.

Both the PCV20 and PCV21 vaccines were licensed based on non-inferiority in immunogenicity, assessed through opsonophagocytic activity (OPA) comparisons, an accepted, although somewhat arbitrary, surrogate measure for licensure of new vaccines. Moreover, previous experience revealed that while randomised controlled trials of vaccines found them non-inferior to comparators for some specific serotypes, real-world effectiveness/impact was lower (i.e. serotype 3 in PCV13 vs PCV7, which demonstrated significantly higher immunogenicity, but no effectiveness [14]), while for others that did not meet non-inferiority, real-world effectiveness/impact was significant (i.e. serotypes 4, 6B and 9V in PCV13 vs PCV7 [15]). Thus, for all newly included serotypes, real-world effectiveness and impact remain unknown and will need to be evaluated post-licensure. While the inclusion of a new serotype in a PCV vaccine will likely provide protection, we cannot yet determine if, and to what extent, effectiveness will be established for each serotype and vaccine. At this stage, we can only refer to the number and identity of serotypes covered by each vaccine and assess their potential impact based on the currently circulating serotypes identified through our comprehensive population-based surveillance data.

Serotype 3, covered by both the PCV20 and PCV21 vaccines, is a clinically important serotype to adults, associated with significant morbidity. Notably, all previously licensed vaccines containing this serotype have shown limited or no effectiveness and no impact against this serotype. We and others have reported persistent high incidence over time and across age groups, regardless of vaccination [3]. While serotype 3 is included in both vaccines, the potential differential effectiveness of PCV20 and PCV21 against this serotype remains to be determined.

Serotype 8, the second most common IPD-causing serotype in older adults, is also covered by both the PCV20 and PCV21 vaccines but not by the PCV13 vaccine. While this serotype did not meet the non-inferiority criterion compared with the PPSV23 vaccine in an immunogenicity study of PCV20-vaccinated naïve adults [16], real-world effectiveness data are needed to determine its protection.

All the VT20-only serotypes are covered by the PCV13 vaccine and thus their circulation was expected to be suppressed following widespread vaccination in children. In our study population, serotype 14 persisted and even somewhat increased following the COVID-19 pandemic period. A recently published report from Belgium also reported the reemergence of serotype 14 in the late/post-pandemic period [17]. It remains to be observed whether this increase reflects a temporary rebound following the decrease observed during the initial COVID-19 pandemic period or whether it will persist in the coming years. Serotype 19F was the second most common among the VT20-only group and showed stable incidence throughout the study period. Interestingly, serotypes 19F and 19A are reported to persist in most surveillance reports, despite high vaccination rates, often even with adult vaccination programmes [3,18-20]. Multiple countries, including Canada, Spain, the United Kingdom and the US, have reported a rise in serotype 4 incidence post PCV13 vaccine introduction [2,21-23]. In the US, this increase has been noted in certain regions, especially among adults with specific underlying risk factors for IPD, such as homelessness [21]. Yet, in our nationwide surveillance, we did not observe any increase in serotype 4 in the late/post COVID-19 pandemic years.

The VT20-only serotype group needs to be monitored very closely if the PCV21 vaccine is used, since the disease rates among older adults following implementation of the PCV21 vaccine will be strictly dependent on PCV20 or PCV15 vaccination coverage of infants. In Israel, vaccination of older adults with PPV23 exceeded 70% (Israel MOH data) until it was replaced by the PCV20 vaccine in July 2023 and became available in early 2024. Consequently, it is still too early to expect any measurable impact on serotype distribution. If a switch to the PCV21 vaccine is made, the protective impact exerted by the PPV23 vaccine against the PCV20-only serotypes, although not ideal (being a non-conjugate polysaccharide vaccine) will be terminated. Thus, the incidence of IPD caused by this serotype group may potentially rise among older adults.

Of the VT21-only serotypes, 16F was the most common serotype (IR: 1.1/100,000), but other serotypes (9N, 15, 23A, 23B, 24F, 31 and 35B) were also common, with incidences exceeding 0.3 per 100,000 population, each. If the PCV21 vaccine is not introduced, these circulating serotypes may further emerge and should also be closely monitored.

With both the PCV20 and PCV21 vaccines soon to be available in Israel, determining an optimal vaccination policy for adults presents a challenge as both offer substantial protection against the serotypes responsible for IPD in this population. According to the latest recommendations from the ACIP, either PCV20 or PCV21 vaccine are now an option in the US for adults aged 19 and older who are currently advised to receive pneumococcal conjugate vaccination, with no prioritisation [9]. Based on our analysis, the VT20-only serotypes accounted for 10.9% of all IPD episodes among older adults in the late/post COVID-19 pandemic period, while the VT21-only serotypes accounted for 25.5%. Although the gap in incidence between the two serotype groups has narrowed over time, VT21-only incidence remained significantly higher than that of VT20-only. Furthermore, following the replacement of the PCV13 with the PCV20 vaccine in Israel's paediatric NIP (in February 2025), additional indirect effects on adult IPD incidence are anticipated. Specifically, PCV20 VT serotypes are expected to decline in the paediatric population leading to reduced transmission to adults. Consequently, the incremental benefit of the PCV21 over the PCV20 vaccine in adults may be further increased. However, the actual magnitude of this indirect effect is expected to be observed only after 1–2 years of paediatric NIP implementation, in real-world impact studies. Moreover, this introduction could also result in an increase in PCV20 NVT serotypes, some of which may not be covered by the PCV21 vaccine. This suggests that the PCV21 vaccine may provide broader coverage in the current epidemiological context in our region. However, as discussed above, IPD serotype dynamics following the introduction of newer vaccines cannot be fully anticipated, making this decision complex. Thus, exploring the potential effect on clinical outcomes, particularly mortality, is also important. We have recently reported that while PCV13 VT serotypes had a substantial mortality burden (case fatality rate (CFR): 21.1%), the additional serotypes in VT20 beyond the PCV13 vaccine coverage (VT20–13) had additional CFR of 16.2%, while the PCV20 NVT serotypes had CFR of 28.5%. Two serotypes with particularly high CFR were 31 and 35B (37% and 38% accordingly), which are among the VT21-only serotypes [24].

Another uncertainty is the future epidemiologic trends of serotypes not covered by either vaccine (NVT serotypes), particularly serotype 2, previously covered by the PPSV23 vaccine, which is no longer in use, as well as other circulating NVT serotypes such as 7B, 6C, 38 and others.

This study has three main limitations. First, as stated above, the potential impact of each of the PCV20 and PCV21 vaccines is not possible to fully predict before their actual implementation. Second, we included only blood and CSF culture-confirmed IPD episodes, which probably resulted in some underestimation of the true IPD burden. However, these inclusion criteria were applied consistently throughout the 15-year surveillance period, allowing for reliable analysis of trends over time and for valid comparisons between vaccine-related serotype groups. Last, the current analysis did not differentiate between serotypes 15B and 15C, reporting both as a single serotype 15B/C. However, the PCV20 vaccine contains the 15B but not 15C polysaccharide and the PCV21 vaccine contains the equivalent of the 15C but not 15B polysaccharide. Despite there being a wide cross protection between these two polysaccharides it does not fully overlap. Furthermore, most surveillance systems, including ours, do not differentiate between the two and report these as 15B/C [5,25,26]. Future real-world studies are likely to provide further insights into the differences between the PCV20 and PCV21 vaccines regarding the protection against serotypes 15B and 15C. Notably, our analysis was limited to IPD; however, these vaccines are also expected to impact non-invasive pneumococcal disease where serotype distribution may differ slightly [27].

## Conclusion

The availability of the new extended-serotype PCVs PCV20 and PCV21 provides additional protection against IPD in adults aged 65 years and older beyond that achieved by the PCV13 vaccine. Currently, the PCV21 vaccine appears to offer a broader potential coverage in Israel. Furthermore, the introduction of the PCV20 vaccine to the paediatric NIP, may further enhance this benefit. However, the ongoing changes in IPD epidemiology following the COVID-19 pandemic may modify the expected clinical impact. Furthermore, the effectiveness of the newly introduced serotypes has yet to be determined. Despite their expanded potential coverage, these vaccines remain selective vaccines. Therefore, the implementation of these vaccines must be accompanied by comprehensive surveillance programmes to monitor pneumococcal disease and transmission patters across all ages.

## Data Availability

All data presented in the manuscript or supplementary material are available from the corresponding author upon reasonable request**.**

## Supplementary Data

461000 Supplementary Material
