# Supplementary material for: Anticipating impact of implementing PCV20 or PCV21 vaccines for older adults in the immunisation programme for invasive pneumococcal disease, using nationwide surveillance data, Israel, 2009 to 2024
Source: Euro Surveill. 2026 Apr 16;31(15):2500589. doi: 10.2807/1560-7917.ES.2026.31.15.2500589 (PMC13090754; doi:10.2807/1560-7917.ES.2026.31.15.2500589)
Supplement: Supplementary Material [file 25-00589_REGEV-YOCHAY_Supplement.pdf]

Supplementary Tables and Figures: distribution of invasive pneumococcal disease (IPD) by serotype groups and the temporal trends of selected individual serotypes, Israel, 2009-2024

This supplementary material is hosted by *Eurosurveillance* as supporting information alongside the article ‘Anticipating impact of implementing PCV20 or PCV21 vaccines for older adults in the immunisation programme for invasive pneumococcal disease, using nationwide surveillance data, Israel, 2009 to 2024', on behalf of the authors, who remain responsible for the accuracy and appropriateness of the content.

The same standards for ethics, copyright, attributions and permissions as for the article apply. Supplements are not edited by *Eurosurveillance* and the journal is not responsible for the maintenance of any links or email addresses provided therein.

Supplementary Table S1. Annual number, incidence rates (per 100,000), proportions (%) and corresponding incidence rate ratios (IRRs) of all IPD, and IPD caused by serotypes included in PCV13, PCV20, PCV21, and Non-VT, as well as serotypes unique to PCV20 and PCV21 (VT20-only and VT21-only), among adults aged ≥65 years, Israel, 2009-2024

Year of diagnosis	**All-IPD** IR (No. cases)	**VT13 IPD** IR (No. cases)	**VT13 IPD** Proportion % of all IPD	**VT20 IPD** IR (No. cases)	**VT20 IPD** Proportion % of all IPD	**VT21 IPD** IR (No. cases)	**VT21 IPD** Proportion % of all IPD	**Non-VT[a] IPD** IR (No. cases)	**Non-VT[a] IPD** Proportion % of all IPD	**IRR [VT21/VT20] (95%CI)**	**VT20-only[b] IPD** IR (No. cases)	**VT20-only[b] IPD** Proportion % of all IPD	**VT21-only[c] IPD** IR (No. cases)	**VT21-only[c] IPD** Proportion % of all IPD	**IRR [VT21-only/VT20-only] (95% CI)**
July 2009 - June 2010	28.25 (n=210)	18.56 (n=138)	65.71%	21.25 (n=158)	75.24%	14.66 (n=109)	51.90%	2.69 (n=20)	9.52%	**0.69 (0.54–0.88)**	9.95 (n=74)	35.24%	3.36 (n=25)	11.90%	**0.34 (0.21–0.53)**
July 2010 - June 2011	31.77 (n=244)	16.53 (n=127)	52.05%	22.00 (n=169)	69.26%	17.97 (n=138)	56.56%	2.99 (n=23)	9.43%	**0.82 (0.65–1.02)**	9.76 (n=75)	30.74%	5.73 (n=44)	18.03%	**0.59 (0.40–0.85)**
July 2011 - June 2012	31.29 (n=250)	16.64 (n=133)	53.20%	21.40 (n=171)	68.40%	18.40 (n=147)	58.80%	3.50 (n=28)	11.20%	**0.86 (0.69–1.07)**	8.51 (n=68)	27.20%	5.51 (n=44)	17.60%	**0.65 (0.44–0.95)**
July 2012 - June 2013	24.64 (n=205)	8.90 (n=74)	36.10%	13.58 (n=113)	55.12%	16.95 (n=141)	68.78%	3.73 (n=31)	15.12%	**1.25 (0.97–1.60)**	3.13 (n=26)	12.68%	6.49 (n=54)	26.34%	**2.07 (1.30–3.31)**
July 2013 - June 2014	25.17 (n=218)	8.89 (n=77)	35.32%	13.97 (n=121)	55.50%	16.97 (n=147)	67.43%	3.35 (n=29)	13.30%	**1.21 (0.95–1.55)**	4.04 (n=35)	16.06%	7.04 (n=61)	27.98%	**1.74 (1.15–2.64)**
**Early PCV7/13 period** (07/2009 - 06/2014)	**28.11 (n=1127)**	**13.70 (n=549)**	**48.71%**	**18.26 (n=732)**	**64.95%**	**17.01 (n=682)**	**60.51%**	**3.27 (n=131)**	**11.62%**	**0.93 (0.84–1.03)**	**6.94 (n=278)**	**24.67%**	**5.69 (n=228)**	**20.23%**	**0.82 (0.69-0.98)**
July 2014 - June 2015	23.96 (n=216)	6.43 (n=58)	26.85%	12.76 (n=115)	53.24%	17.42 (n=157)	72.69%	4.10 (n=37)	17.13%	**1.37 (1.07–1.74)**	2.11 (n=19)	8.80%	6.77 (n=61)	28.24%	**3.21 (1.92–5.37)**
July 2015 - June 2016	24.06 (n=226)	6.60 (n=62)	27.43%	12.99 (n=122)	53.98%	17.04 (n=160)	70.80%	3.41 (n=32)	14.16%	**1.31 (1.04–1.66)**	2.66 (n=25)	11.06%	6.71 (n=63)	27.88%	**2.52 (1.59–4.01)**
July 2016 - June 2017	27.29 (n=267)	6.75 (n=66)	24.72%	14.11 (n=138)	51.69%	19.22 (n=188)	70.41%	4.50 (n=44)	16.48%	**1.36 (1.09–1.70)**	2.35 (n=23)	8.61%	7.46 (n=73)	27.34%	**3.17 (1.99–5.07)**
July 2017 - June 2018	24.47 (n=249)	6.00 (n=61)	24.50%	14.25 (n=145)	58.23%	18.18 (n=185)	74.30%	3.93 (n=40)	16.06%	**1.28 (1.03–1.59)**	1.47 (n=15)	6.02%	5.41 (n=55)	22.09%	**3.68 (2.08–6.51)**
July 2018 - June 2019	23.87 (n=252)	4.17 (n=44)	17.46%	11.27 (n=119)	47.22%	17.71 (n=187)	74.21%	3.79 (n=40)	15.87%	**1.57 (1.25–1.98)**	1.52 (n=16)	6.35%	7.96 (n=84)	33.33%	**5.24 (3.07–8.94)**
**Late PCV13 period** (07/2014 - 06/2019)	**24.73 (n=1210)**	**5.95 (n=291)**	**24.05%**	**13.06 (n=639)**	**52.81%**	**17.93 (n=877)**	**72.48%**	**3.95 (n=193)**	**15.95%**	**1.37 (1.24-1.52)**	**2.00 (n=98)**	**8.10%**	**6.87 (n=336)**	**27.77%**	**3.43 (2.74-4.30)**

July 2019 - June 2020	18.39 (n=201)	4.48 (n=49)	24.38%	9.88 (n=108)	53.73%	13.27 (n=145)	72.14%	2.47 (n=27)	13.43%	**1.34 (1.05–1.72)**	1.74 (n=19)	9.45%	5.12 (n=56)	27.86%	**2.94 (1.75–4.95)**
July 2020 - June 2021	13.03 (n=147)	2.22 (n=25)	17.01% (n=25)	6.47 (n=73)	29.25%	9.31 (n=105)	81.63%	1.86 (n=21)	14.29%	**1.44 (1.07–1.94)**	0.98 (n=11)	7.48%	3.81 (n=43)	29.25%	**3.89 (2.00–7.54)**
**COVID-19 Period** (07/2019 – 06/2021)	**15.67 (n=348)**	**3.33 (n=74)**	**21.26%**	**8.15 (n=181)**	**52.01%**	**11.26 (n=250)**	**71.84%**	**2.16 (n=48)**	**13.79%**	**1.38 (1.14-1.67)**	**1.35 (n=30)**	**8.62%**	**4.46 (n=99)**	**28.45%**	**3.30 (2.20-4.97)**
July 2021 - June 2022	22.34 (n=260)	6.10 (n=71)	27.31%	11.94 (n=139)	53.46%	16.24 (n=189)	72.69%	3.52 (n=41)	15.77%	**1.36 (1.09–1.69)**	1.55 (n=18)	6.92%	5.84 (n=68)	26.15%	**3.77 (2.24–6.33)**
July 2022 - June 2023	24.44 (n=308)	7.46 (n=94)	30.52%	12.62 (n=159)	51.62%	16.27 (n=205)	66.56%	4.28 (n=54)	17.53%	**1.29 (1.05–1.59)**	2.38 (n=30)	9.74%	6.03 (n=76)	24.68%	**2.53 (1.66–3.87)**
July 2023 - June 2024	22.90 (n=300)	6.64 (n=87)	29.00%	11.53 (n=151)	50.33%	14.81 (n=194)	64.67%	3.97 (n=52)	17.33%	**1.28 (1.04–1.59)**	2.75 (n=36)	12.00%	6.03 (n=79)	26.33%	**2.19 (1.48–3.25)**
**Last two years post COVID-19** (07/2022 - 06/2024)	**23.65 (n=608)**	**7.04 (n=181)**	**29.77%**	**12.06 (n=310)**	**50.99%**	**15.52 (n=399)**	**65.62%**	**4.12 (n=106)**	**17.43%**	**1.29 (1.11-1.49)**	**2.57 (n=66)**	**10.86%**	**6.03 (n=155)**	**25.49%**	**2.35 (1.76-3.13)**
**Total stable period[d]**	**24.09 (n=2078)**	**6.29 (n=543)**	**26.13%**	**12.61 (n=1088)**	**52.36%**	**16.98 (n=1465)**	**70.50%**	**3.94 (n=340)**	**16.36%**	**1.35 (1.24-1.46)**	**2.11 (n=182)**	**8.76%**	**6.48 (n=559)**	**26.90%**	**3.07 (2.60-3.63)**

CI - confidence interval; IPD - invasive pneumococcal disease; IR - incidence rate; IRR - incidence rate ratio; No. – number; Non-VT - non-pneumococcal conjugated vaccine serotypes; PCV - pneumococcal conjugated vaccine; VT - pneumococcal conjugated vaccine serotypes

[a] Non-VT includes all serotypes not included in either PCV20 or PCV21

[b] VT20-only includes the serotypes included in PCV20 but not in PCV21

[c] VT21-only includes the serotypes included in PCV21 but not in PCV20

[d] Total stable period: 2014-2024, excluding COVID-19 period (2019-21)

Supplementary Table S2. Incidence rates, vaccine group affiliation, and proportion of all IPD episodes for the most common individual serotypes among adults aged ≥65 years, Israel, 2022-2024

Serotype	Vaccine group	Incidence rate (No. of cases)	% of IPD
3	VT20&21[a]	3.03 (n=78)	12.83%
8	VT20&21[a]	1.87 (n=48)	7.89%
14	VT20-only[b]	1.52 (n=39)	6.41%
19A	VT20&21[a]	1.36 (n=35)	5.76%
16F	VT21-only[c]	1.09 (n=28)	4.61%
9N	VT21-only[c]	0.82 (n=21)	3.45%
15A	VT21-only[c]	0.82 (n=21)	3.45%
7B	Non-VT[d]	0.82 (n=21)	3.45%
24F	VT21-only[c]	0.78 (n=20)	3.29%
6C	Non-VT[d]	0.74 (n=19)	3.13%
12F	VT20&21[a]	0.70 (n=18)	2.96%
23A	VT21-only[c]	0.70 (n=18)	2.96%

IPD - invasive pneumococcal disease; Non-VT - non-pneumococcal conjugated vaccine serotypes; VT - pneumococcal conjugated vaccine serotypes

[a] VT20&21 includes the serotypes covered by both PCV20 and PCV21

[b] VT20-only includes the serotypes included in PCV20 but not in PCV21

[c] VT21-only includes the serotypes included in PCV21 but not in PCV20

[d] Non-VT includes all serotypes not included in either PCV20 or PCV21

Supplementary Figure S1. Serotype Coverage Comparison of PCV13, PCV20, and PCV21 Vaccines

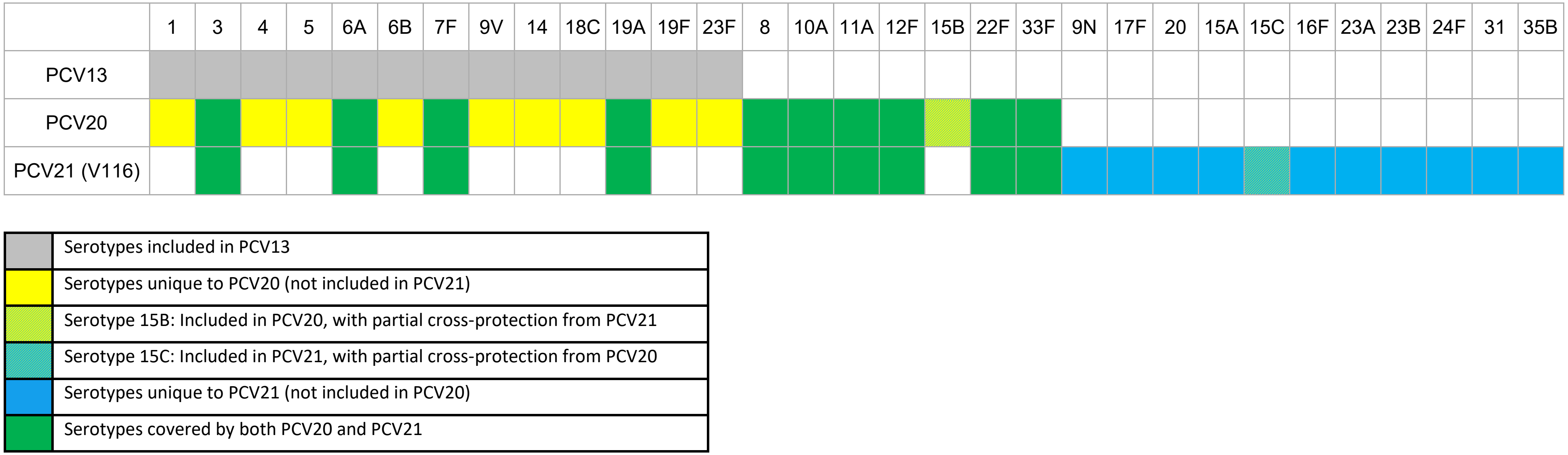

PCV - pneumococcal conjugated vaccine serotypes

Supplementary Figure S2a. Annual Incidence of serotype 3 IPD per 100,000 in older adults (≥65y), Israel, 2009-2024 (n=388 IPD episodes)

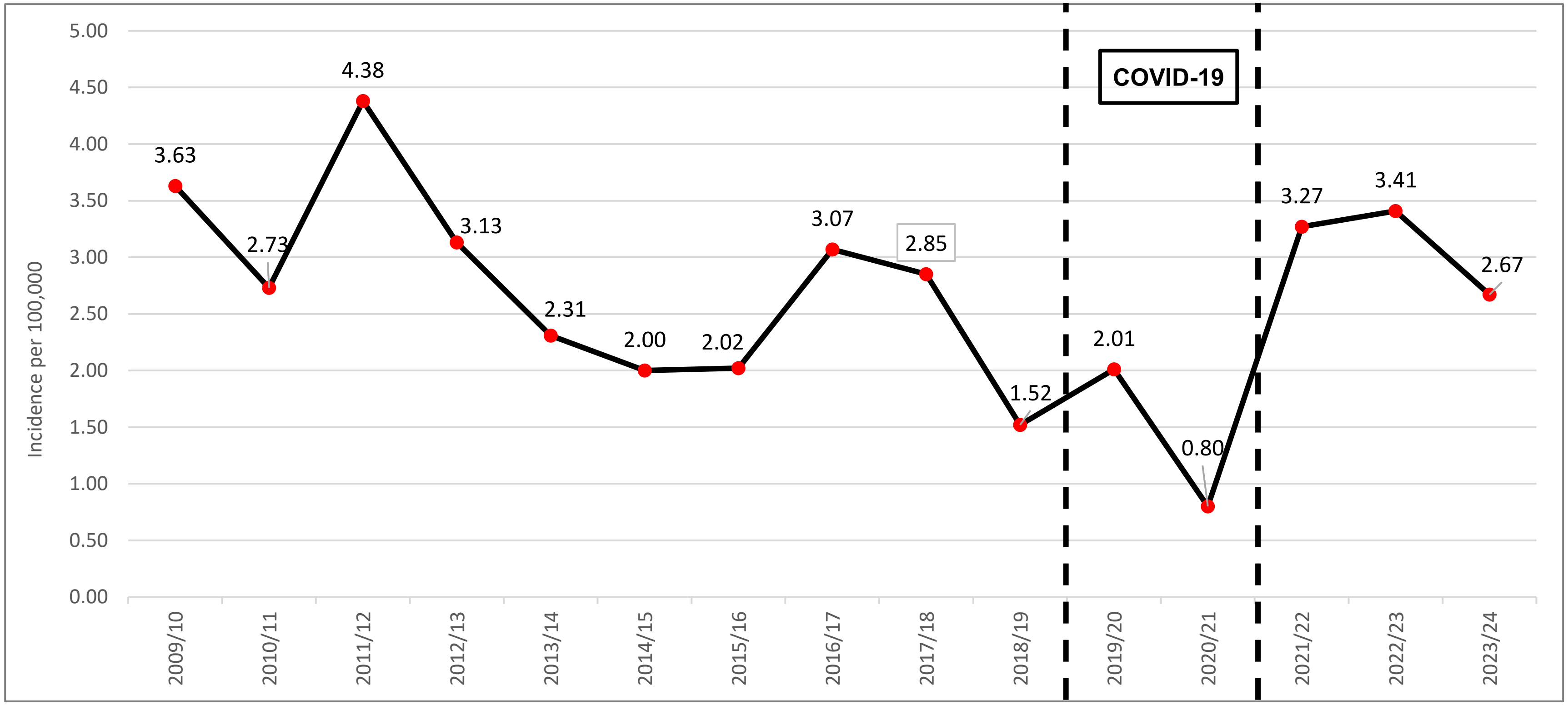

IPD – invasive pneumococcal disease

Supplementary Figures S2b-d. Annual Incidence of serotype 3 IPD per 100,000 in Older Adults by Age groups (65-74, 75-84, and ≥85 years), Israel, 2009-2024

b. Age 65-74

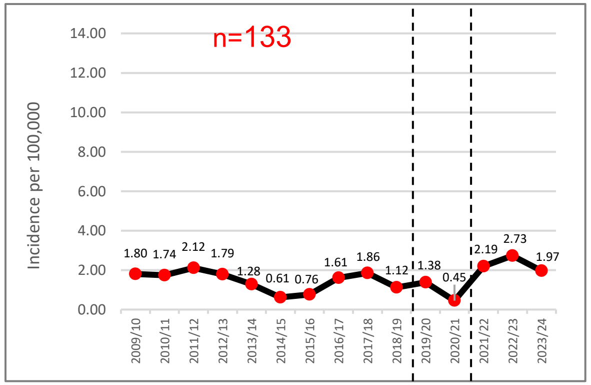

c. Age 75-84

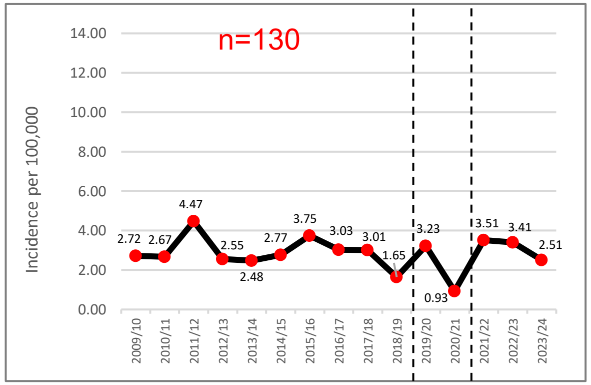

d. Age ≥85

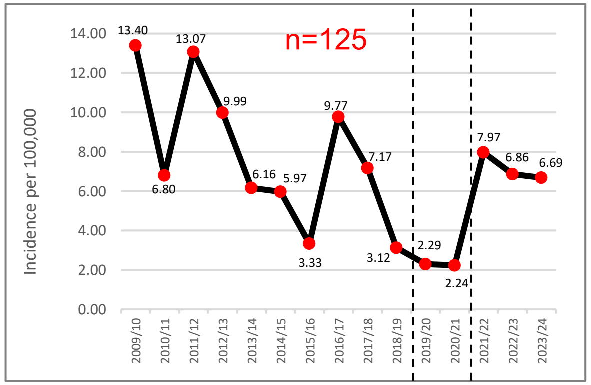

IPD – invasive pneumococcal disease

Supplementary Figure S3a. Annual Incidence of serotype 8 IPD per 100,000 in older adults (≥65y), Israel, 2009-2024 (n=254 IPD episodes)

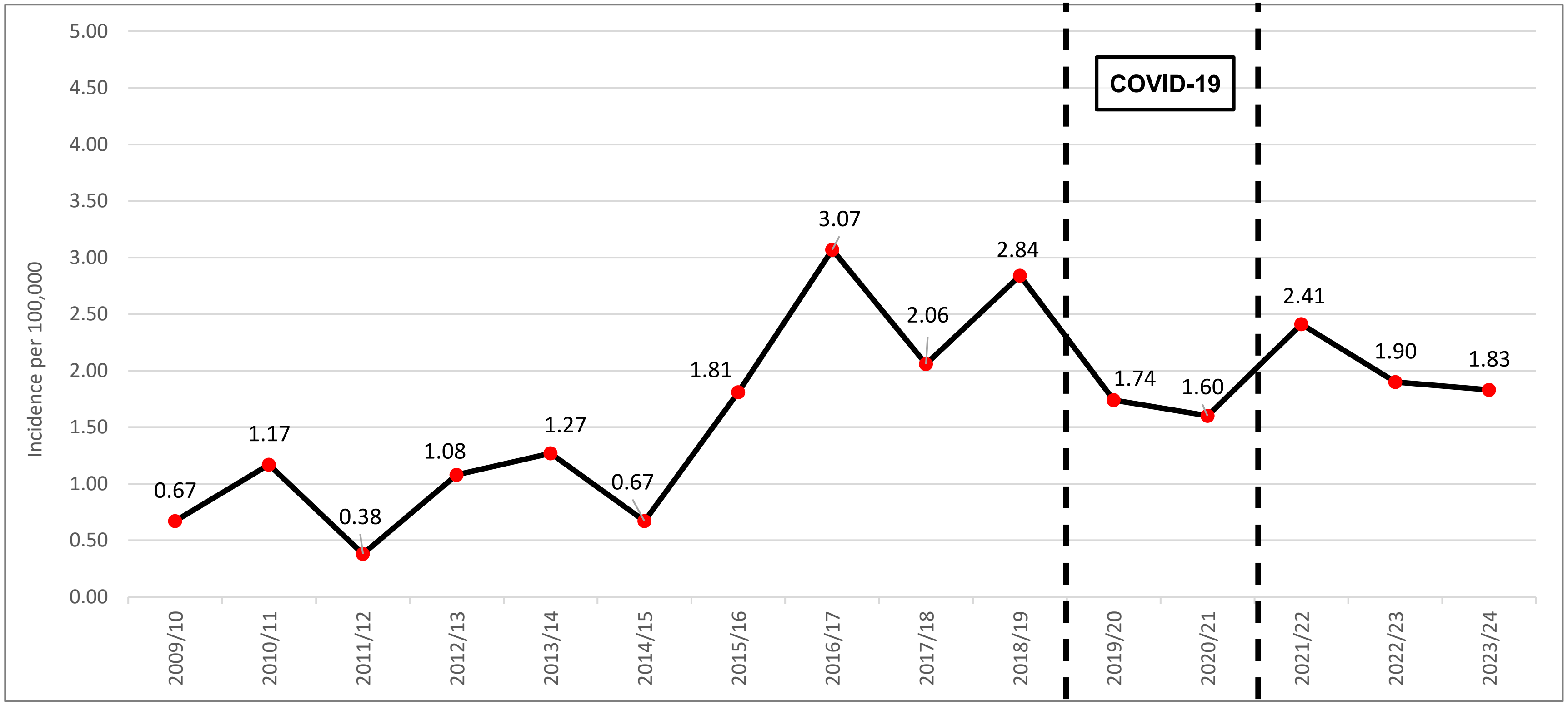

IPD – invasive pneumococcal disease

Supplementary Figures S3b-d. Annual Incidence of serotype 8 IPD per 100,000 in Older Adults by Age groups (65-74, 75-84, and ≥85 years), Israel, 2009-2024

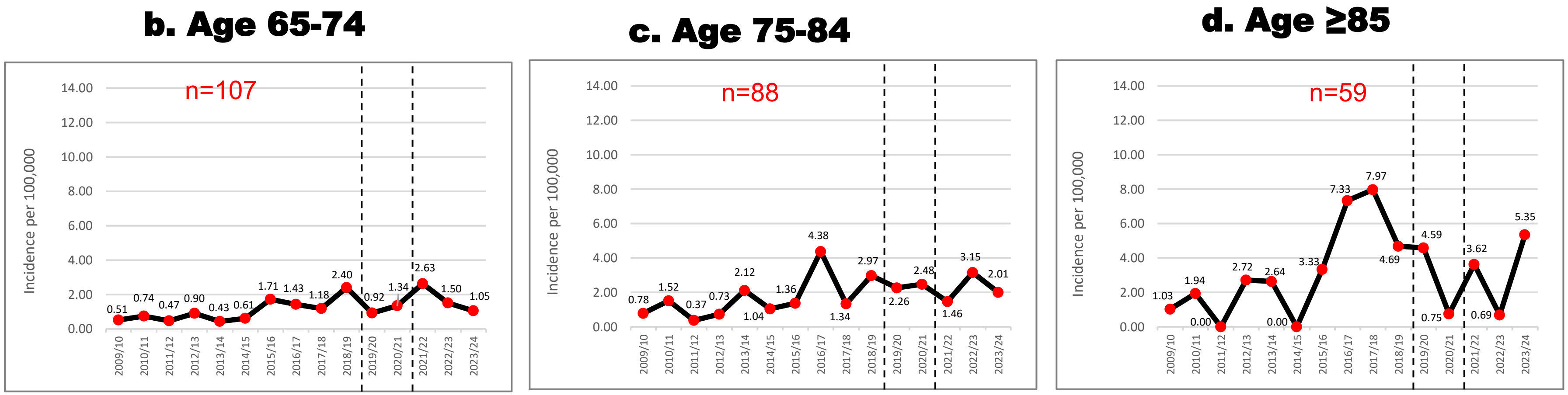

IPD – invasive pneumococcal disease

Supplementary Figure S4a. Annual Incidence of serotype 14 IPD per 100,000 in older adults (≥65y), Israel, 2009-2024 (n=153 IPD episodes)

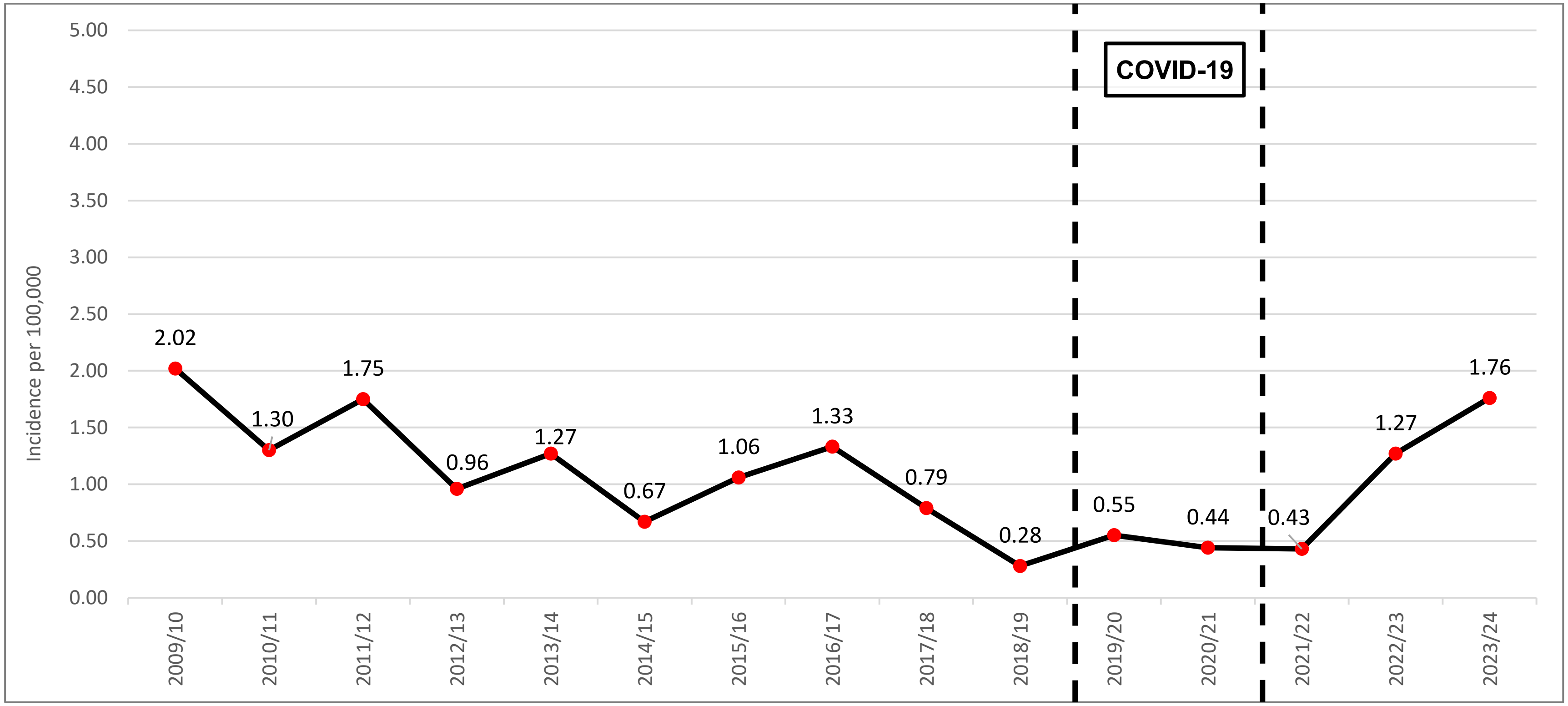

IPD – invasive pneumococcal disease

Supplementary Figures S4b-d. Annual Incidence of serotype 14 IPD per 100,000 in Older Adults by Age groups (65-74, 75-84, and ≥85 years), Israel, 2009-2024

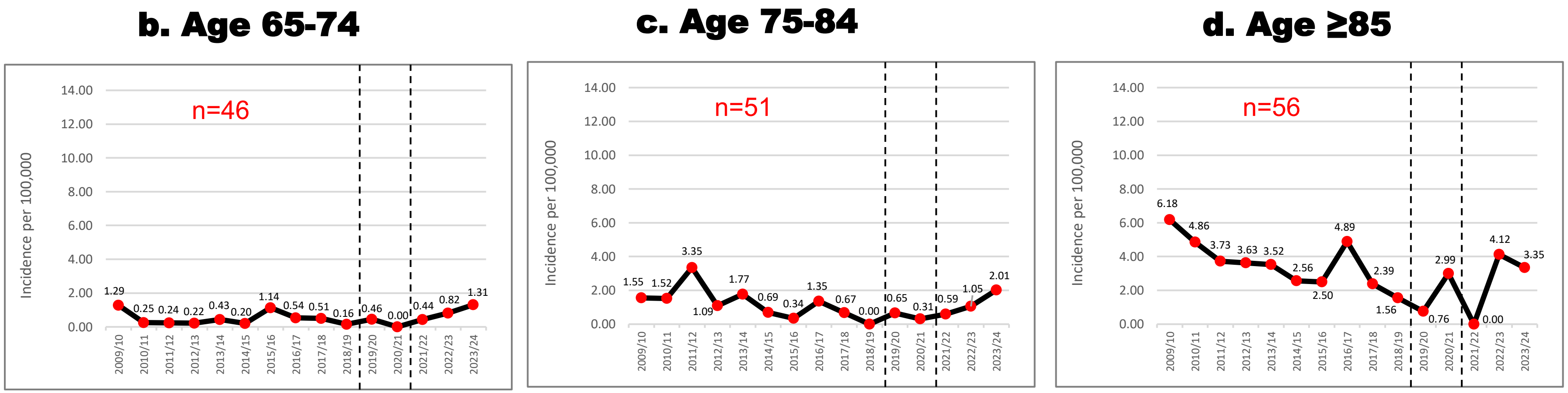

IPD – invasive pneumococcal disease

Supplementary Figure S5a. Annual Incidence of serotype 19A IPD per 100,000 in older adults (≥65y), Israel, 2009-2024 (n=221 IPD episodes)

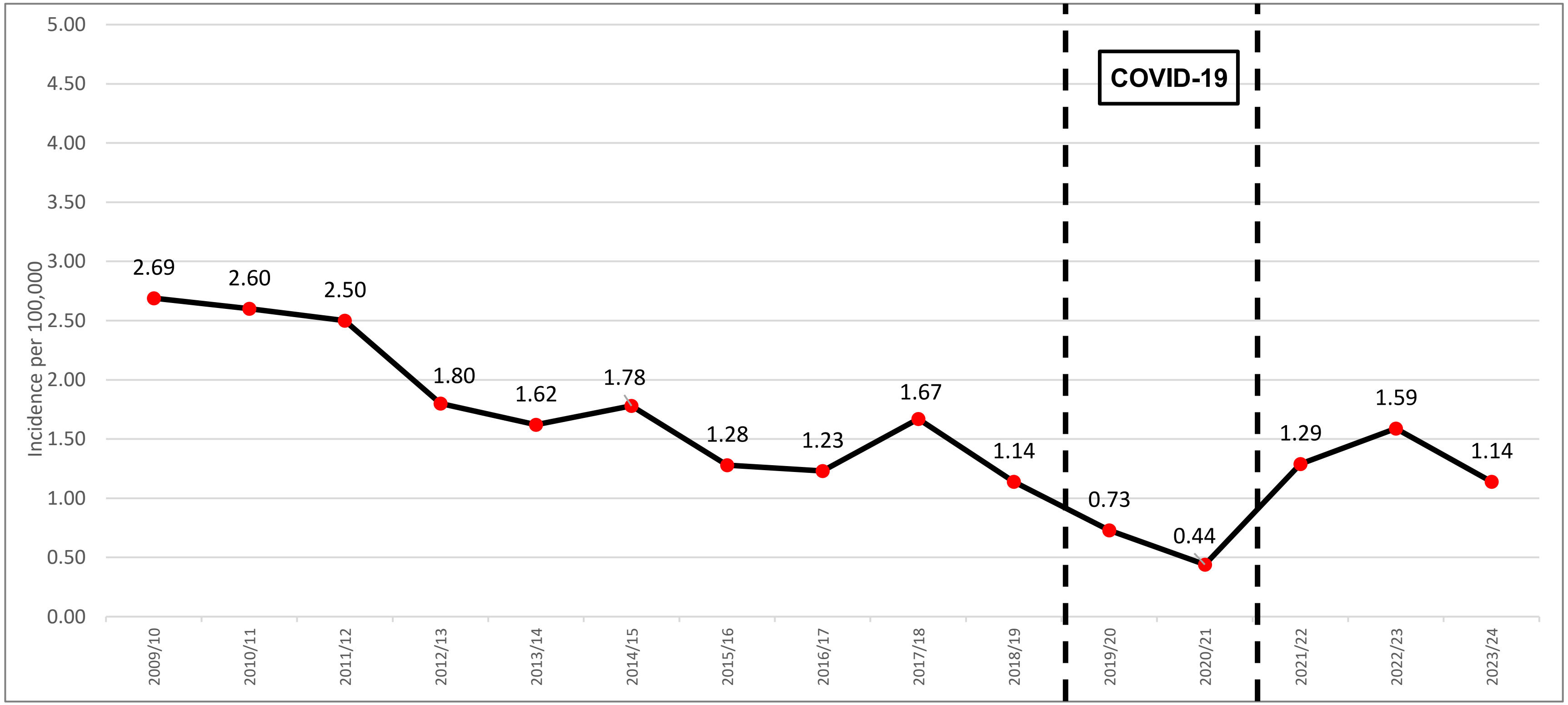

IPD – invasive pneumococcal disease

Supplementary Figures S5b-d. Annual Incidence of serotype 19A IPD per 100,000 in Older Adults by Age groups (65-74, 75-84, and ≥85 years), Israel, 2009-2024

b. Age 65-74

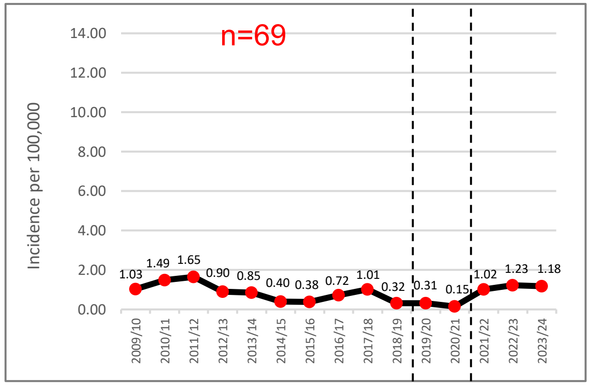

c. Age 75-84

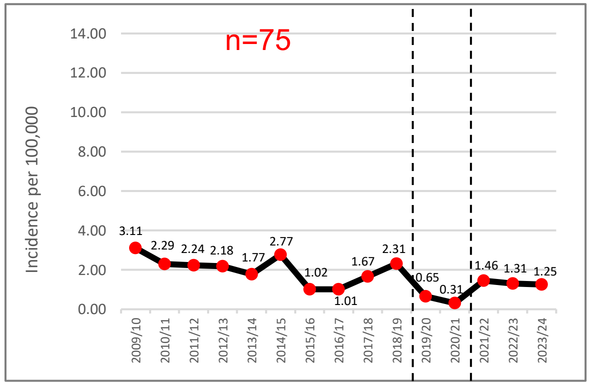

d. Age ≥85

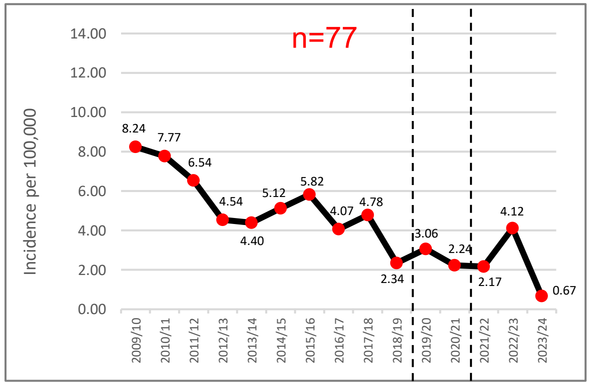

IPD – invasive pneumococcal disease

Supplementary Figure S6a. Annual Incidence of serotype 16F IPD per 100,000 in older adults (≥65y), Israel, 2009-2024 (n=193 IPD episodes)

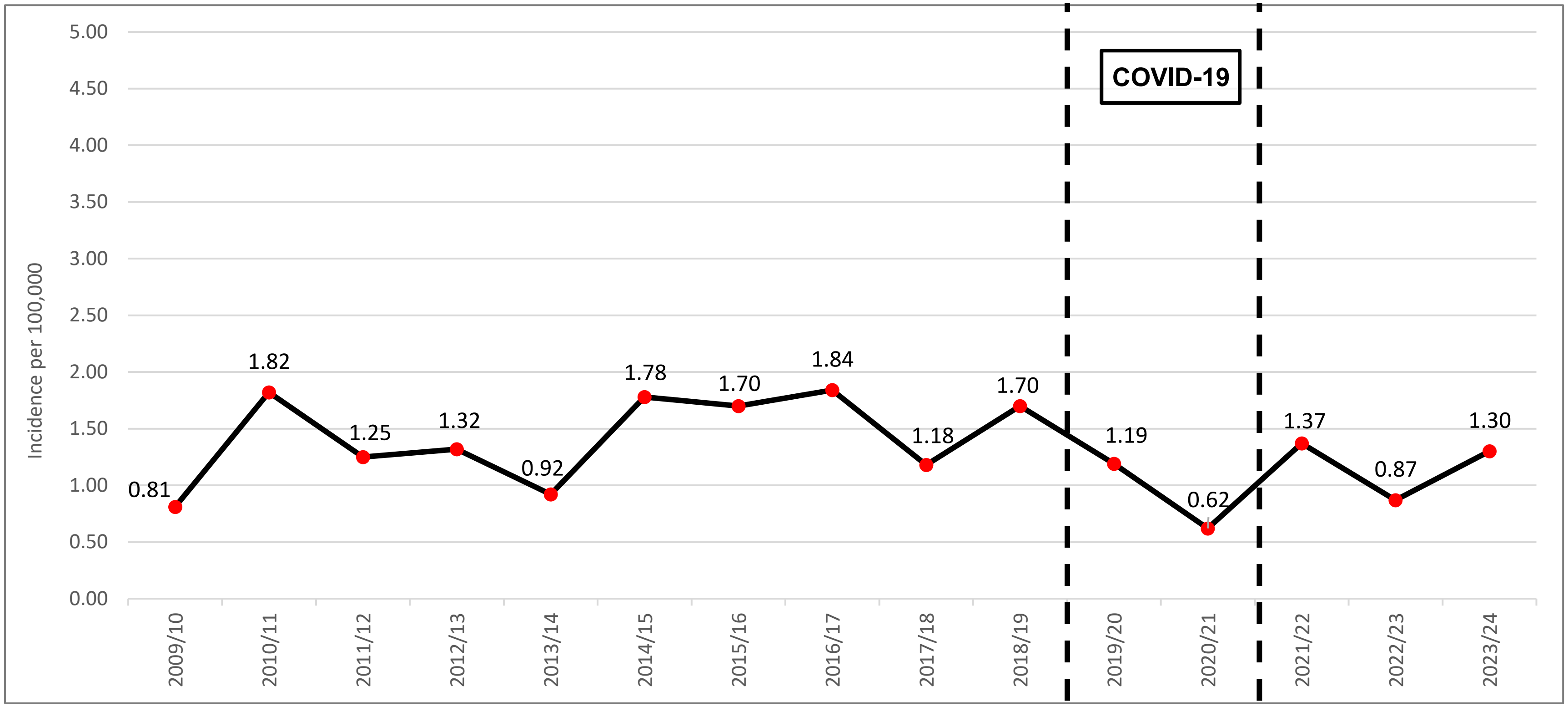

IPD – invasive pneumococcal disease

Supplementary Figures S6b-d. Annual Incidence of serotype 16F IPD per 100,000 in Older Adults by Age groups (65-74, 75-84, and ≥85 years), Israel, 2009-2024

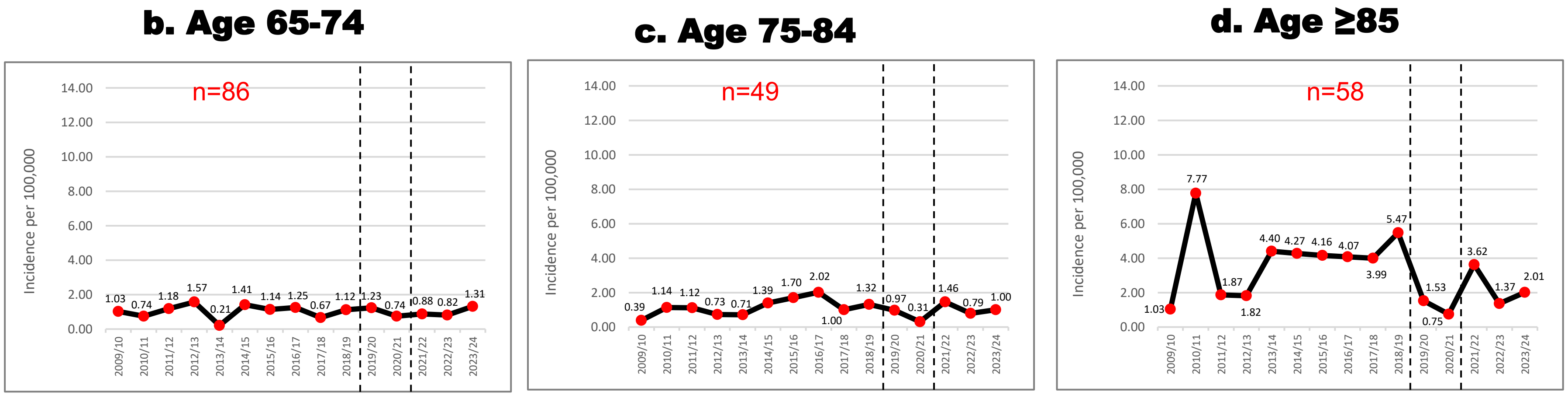

IPD – invasive pneumococcal disease

Supplementary Figure S7. Annual Incidence of serotype 19F IPD per 100,000 in older adults (≥65y), Israel, 2009-2024 (n=77 IPD episodes)

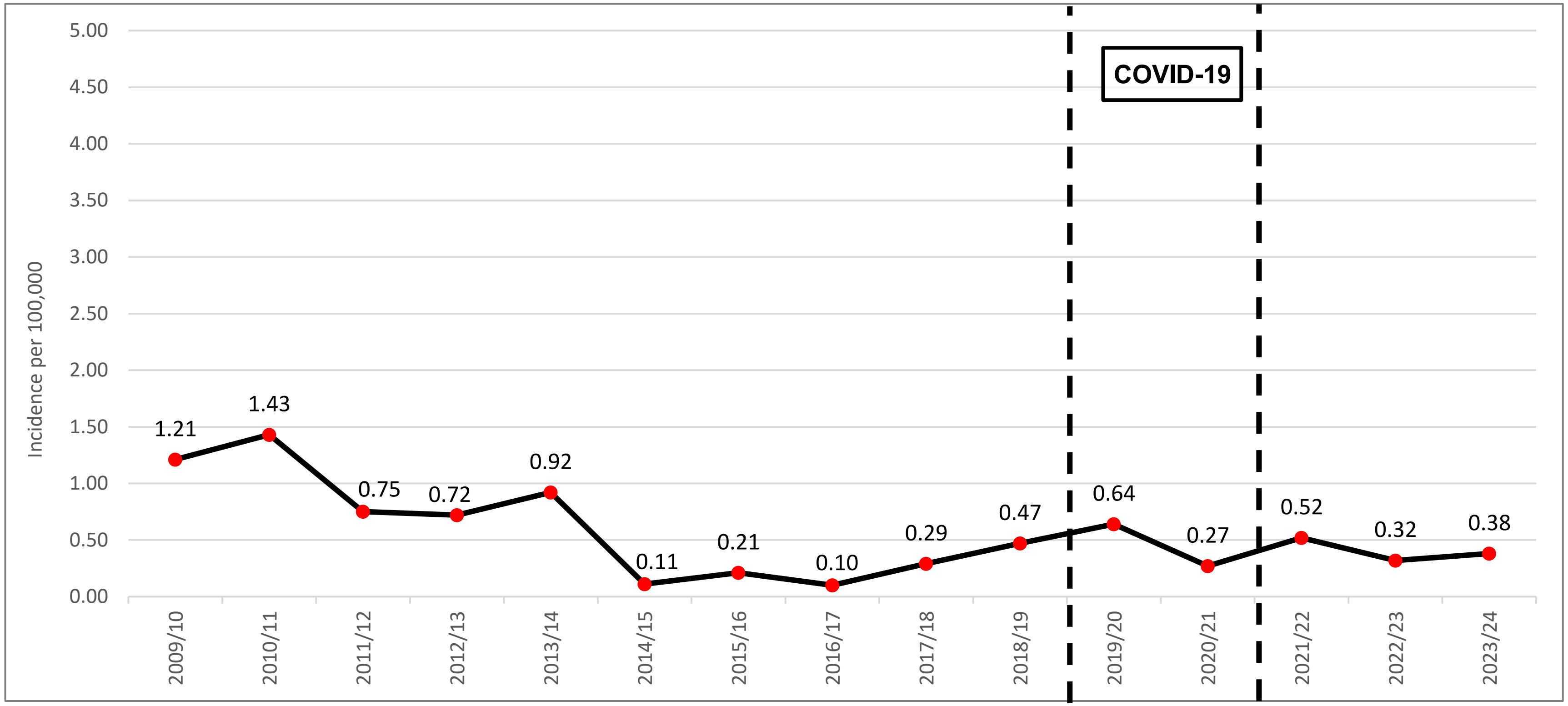

IPD – invasive pneumococcal disease

Supplementary Figure S8. Annual Incidence of serotype 4 IPD per 100,000 in older adults (≥65y), Israel, 2009-2024 (n=21 IPD episodes)

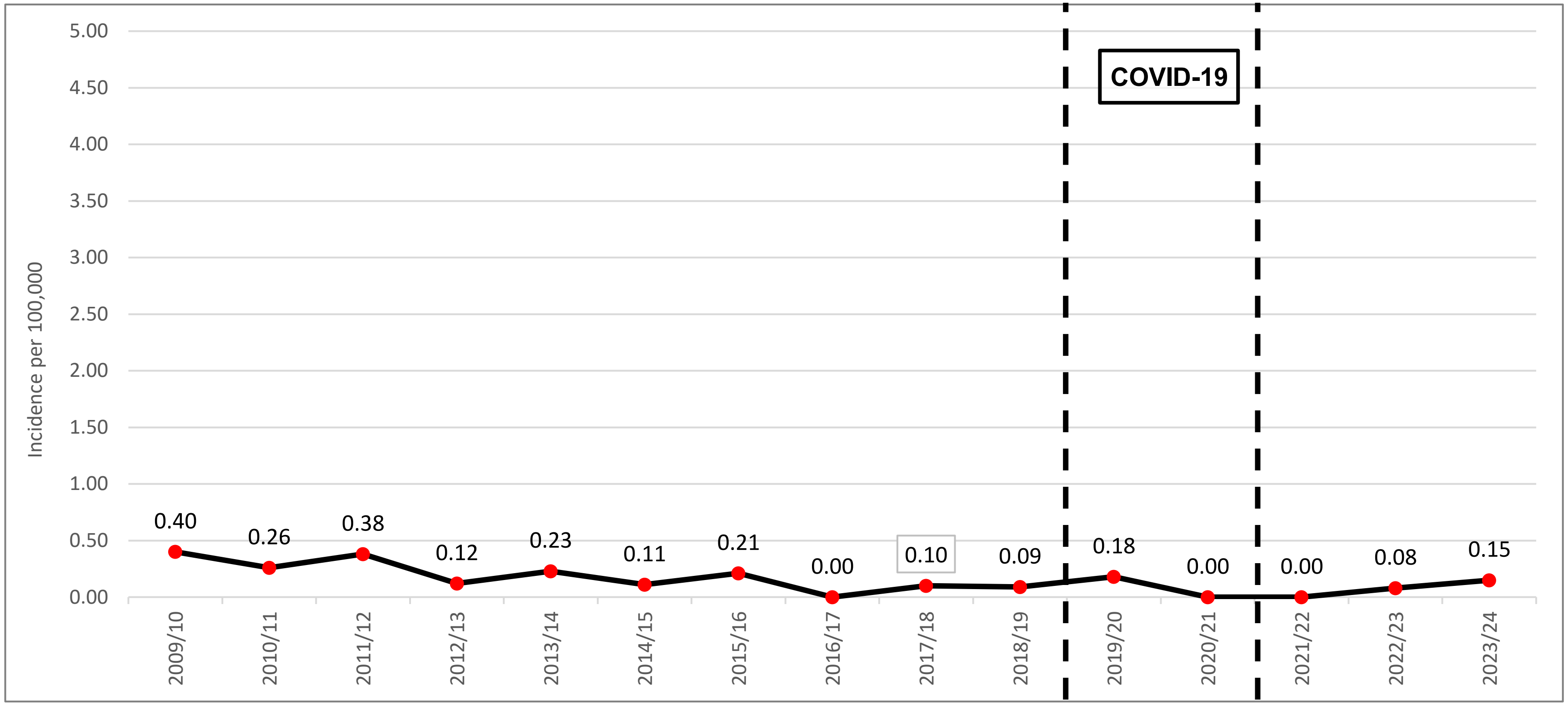

IPD – invasive pneumococcal disease
